# A Link between Parental Psychopathology and Preschool Depression: Take Care of Parents to Take Care of Children

**DOI:** 10.3390/children10010150

**Published:** 2023-01-12

**Authors:** Melania Martucci, Franca Aceti, Nicoletta Giacchetti, Veronica Scarselli, Carla Sogos

**Affiliations:** 1 Child Neuropsychiatry Unit, Department of Human Neuroscience, Sapienza University of Rome, 00185 Rome, Italy; 2 Post-Partum Disorders Unit, Department of Human Neuroscience, Sapienza University of Rome, 00185 Rome, Italy

**Keywords:** preschool depression, children, parents

## Abstract

There is a lot of evidence in the literature showing that early-onset depression determines an emotional and cognitive vulnerability for psychiatric disorders in subsequent years. AIMS: The first aim of this outcome research was to analyze the impact of parental support treatment in a sample of depressed preschool children divided into two groups of comparison (under-reactive and over-reactive) through evolution in the Clinical Global Impression (CGI). The second aim was to analyze the correlation between the presence of parental psychopathology and the severity of children’s disorders. METHODS: Our clinical sample consisted of 32 preschool-age children with a final diagnosis of MDD. The children’s assessment included a psychiatric assessment to establish a diagnosis of MDD, confirmed by means of a semi-structured interview, which was administered again one month after the end of parental treatment. All the parents began a six-month parent training treatment conducted by experienced child psychiatrists, whereas children were not treated. During this period, the Clinical Global Impression Scale (CGI) was filled out monthly in order to observe the evolution of the children’s disorders. Results: Post-hoc tests showed a significant difference from before the treatment to after the treatment only in the over-reactive group (*p* = 0.00). Regarding parental psychiatric disorders, in the over-reactive group, only 3 children had no parents with psychopathology. In the under-reactive group, no child lacked a parent with psychopathology. Conclusion: Parent training treatment seems to be a valid intervention to improve preschool depression, especially in over-reactive groups, and to prevent dysfunctional parental styles connected to parental psychopathology.

## 1. Introduction

There is a lot of evidence in the literature demonstrating that early-onset depression determines an emotional and cognitive vulnerability for psychiatric disorders in subsequent years in the absence of psychosocial protective factors and appropriate therapeutic support. In fact, a follow-up of preschool samples provides evidence of continued risk for depression, at least into the early school years. Considering heterotypic continuities, links with three other disorder groupings (anxiety disorders, alcohol and substance use, and conduct disorders) seem to be very frequent [1]. Furthermore, preschoolers with baseline depression displayed the highest likelihood of subsequent depression 12 and/or 24 months later compared with preschoolers with no baseline disorder and with those suffering from other psychiatric disorders. As regards prevalence, studies applying the Diagnostic and Statistical Manual of mental disorders-5 (DSM 5) criteria, typically assessed with clinical interviews, reveal a low prevalence of Major Depressive Disorder (MDD) during preschool years (2%) and possibly higher prevalence in middle to late childhood and early adolescence (5–8%) [2,3]. The DSM-5 does not distinguish between childhood and adult forms of depression. The same core symptoms include sadness/irritability, anhedonia, concentration difficulty, guilt, recurrent thoughts about death, fatigue, and changes in appetite. A large body of literature has focused on identifying specific diagnostic criteria for preschool depression [4]. This evidence suggests that anhedonia is the most specific symptom in preschoolers [5]. Moreover, it marked a more severe subtype of preschool depression [6]. Research in this field suggests age-specific death preoccupation symptoms (e.g., “persistent choice of play themes with death or suicide”) [6]. Pathological guilt [7] and irritability [8] are part of the core symptoms of preschool depression. Pathological guilt is considered an intense feeling of guilt following a transgression, even for situations for which the child is not responsible [7]. Instead, irritability consists of low frustration tolerance with anger and temper outbursts. Data from a large community sample of preschoolers show that irritability measured at the age of 3 is predictive of both Depression and Oppositional Defiant Disorder (ODD) at the age of 6. Moreover, the same sample showed greater functional impairment at the age of 9 [9]. Sleep disturbances and increased fatigue are common symptoms of preschool depression. More specifically, sleep onset latency and the child’s difficulties sleeping alone are considered risk factors for preschool depression [10]. The validity of these markers was based on parent reports of depressive symptoms, although depressive behavior had been observed during play sessions or parent-child interaction tasks. In this regard, a study by Luby et al. (2006) showed that depressed preschoolers showed anhedonia, more avoidance, and negative feelings than healthy controls. This study reported that depressed preschoolers without externalizing disorders appeared as the only depressed subgroup significantly distinguishable from healthy preschoolers. Hence, the presence of externalizing disorders in addition to depression may reduce impairments in specific areas of the functioning [11] sample. 

Moreover, it is important to detect the presence of several risk factors in order to consider the clinical implications, prognosis, and treatment of depressed preschoolers. Additionally, causes such as biological, psychological, and social origin, as well as a family predisposition, seem to play a pivotal role. Stressors neurobiologically affect the nervous system as well as the regulation of chemical transmitters (e.g., serotonin, noradrenaline, and dopamine) [12]. Psychosocial risks include family bereavement, separations and conflict, child maltreatment and neglect, and peer conflict and bullying, discouraging interpersonal relationships concurrent to the disease [12]. The literature has related parents’ depression [13,14] to preschool-onset depression in their children [15].

Specific parenting practices, such as lower levels of warm parenting, as well as stressful life events and social adversity, have been associated with Preschool Onset Major Depressive Disorder (PO-MDD) [16,17]. Parental depression is also strongly related to financial stress and lower socio-economic status, which significantly affect PO-MDD’s onset [18]. More precisely, a dyadic mother-child relationship is crucial; together with a lack of care, attention, and empathy, the depression of the mother or of both parents, or the absence of a maternal figure, may lead to the child’s emotional deficiency and depression, especially in the first months of life [17]. If we take into account the first year of life, infants of depressed mothers appear to have fewer mature regulatory behaviors, more negative emotionality, and high levels of cortisol reactivity compared to infants of non-affected controls [18]. They are more likely to develop emotional and behavioral problems, especially when maternal depression persists beyond the first postnatal year [19]. A recent study suggested that reduced sleep time at preschool was associated with moderate to severe maternal depression symptoms. This is a very interesting finding, given the important role played by sleep in children’s cognitive performance and prospective related behavioral and emotional problems [20]. According to the evidence, parenting programs involve the use of a range of techniques in their delivery, including discussion, role play, watching video vignettes, and homework [21]. Recent evidence showed that parental support treatment could improve the emotional and behavioral adjustment of children under three years [22] and of children aged three to 10 years [23]. Several studies pointed to a range of benefits of taking part in a group with other parents [21]. Previous studies suggested a better prognosis for depressed preschoolers with disruptive behaviors and a relevant association between preschool depression and parental psychopathology. The formulated hypothesis is that there is a correlation between psychomotor style and a specific set of symptoms and feelings. Previous studies have argued that psychomotor disturbance represents a specific depressive feature as it occurs in melancholic disorders. It is considered to be a sign characterizing “inhibited” or “agitated” behaviors and motricity of depressed patients [24]. What seems particularly significant is the fact that in all subjects, psychomotor style is in some way altered: with a shift to psychomotor agitation or to psychomotor retardation, or, again, to an oscillation of these two extremes. Psychomotor style thus proved itself a symptom of significant importance for characterizing the two clinical groups, each with specific and coherent underlying affective profiles. The varied clinical expression of depressive disorders in this age range, and the underlying structural organization, are, it is suggested, clarified by the definition of these groups. The direct link between depressed affect and depressive behavior, which is characteristic of preschool-age children [25], is also highlighted in each group. Based on this evidence, we have established our aims in order to increase knowledge in this field. The aim of this outcome research was to analyze the impact of the parental support treatment on a sample of depressed preschool children divided into two groups for comparison (under-reactive and over-reactive) through evolution in the Clinical Global Impression (CGI) [26]. The second aim was to analyze the correlation between the presence of parental psychopathology and the severity of the children’s disorders.

## 2. Materials and Methods

The drafting of this cohort study has been realized according to the guidelines for reporting observational studies: the Strengthening the Reporting of Observational Studies in Epidemiology (STROBE) Statement.

### 2.1. Design

Outcome research.

### 2.2. Participants

The population was recruited from the Department of Human Neurosciences–Child Neuropsychiatry Unit, University of Rome “Sapienza”, from December 2019 to June 2021. From the total population of preschool-age children (4 years, 1 month to 5 years, 11 months-old) referred for non-specific developmental or behavioral problems over an 18-month period, a clinical sample of 32 children and their parents was selected (18 males and 14 females; age mean = 4.5 years old, SD = 0.9). Total IQ, Verbal IQ, and Performance IQ, assessed using the Wechsler Preschool and Primary Scale of Intelligence-III (WPPSI-III) [27], of all selected children fell within the normal range (IQ Tot mean = 102.08, SD = 14.31).

The following exclusion criteria were applied:

(1) intellectual deficiency (full-scale Intellectual Quotient < 70 on the Wechsler Preschool and Primary Scale of Intelligence (WPPSI-III) [27], according to DSM-5 criteria [28];
(2) the presence of any neurological and neurosensorial diseases.

The children were subsequently divided into two groups—under-reactive and over-reactive—following discriminating criteria of prevalent psychomotor style [24,28].

The study was carried out in accordance with the Declaration of Helsinki. Written informed consent was obtained from the parents of all participants.

### 2.3. Tools

***Medical assessment***. A medical history was collected, and neurological and physical examinations were performed on all the children to exclude comorbid medical and neurological conditions. Furthermore, a comprehensive history was taken from the parents, with a specific focus on pinpointing signs and symptoms of depression. Other data that could prove significant for a correct clinical understanding of the depressive disorder was also collected; for instance, the presence of risk factors or protective factors, life events, and past symptomatology. Children were included in the under-reactive group if anhedonia, apathy, or inhibition prevailed. Children with externalizing behaviors were included in the over-reactive group.

***Psychiatric and psychological evaluation of children and their parents.*** The children’s psychiatric diagnoses, including Major Depressive Disorder and psychiatric comorbidity, were established according to the DSM-5 criteria [28] and backed up by means of the semi-structured interview Schedule for Affective Disorders and Schizophrenia for School-Age Children—Present and Lifetime Version [29], a useful tool for the assessment of psychopathology in preschoolers, which was administered to parents by an experienced child psychiatrist. The psychiatrist also filled in the Clinical Global Impression [26,30], a measure of children’s symptom severity, treatment response, and efficacy. The CGI is a 7-point scale that requires the clinician to rate the severity of the patient’s symptoms (from 1 = normal to 7 = extremely ill) at the time of the assessment; it is thus possible to evaluate how much the patient’s symptoms have improved or worsened relative to a baseline state [30].

### 2.4. Interventions

During the children’s baseline evaluation, all parents underwent an individual complete psychiatric evaluation in the Adult Psychiatric Unit of a hospital in Rome. After the psychiatric assessment, all parents began a couple of sections of parental support treatment (six months) conducted by experienced child psychiatrists, while the children were not treated.

The monthly parental support sessions, lasting one hour, were aimed at promoting the parents’ awareness of their children’s emotional needs and at sustaining child self-esteem by using a standardized program. In addition to that, during this period, the clinicians tried to involve all the children’s environmental supports (schoolteachers, extended family, peer groups, etc.). During this period, in order to observe the evolution of the children’s disorders, children of both subgroups were evaluated using the Clinical Global Impression [26,30] before the beginning of the parents’ treatment, during the treatment (every 45 days for a total of four measurements) and one month after the end of the treatment. Moreover, the Schedule for Affective Disorders and Schizophrenia for School-Age Children—Present and Lifetime Version, as suggested by Birmaher et al. (2009) [29], was administered again to parents one month after the end of the parents’ treatment. The children of both groups were thus divided into three outcome subgroups: recovered—DSM-5 criteria for Major Depressive Disorder were not met; partially recovered—diagnosis of Depressive Disorder DSM-5 in partial remission; still depressed—diagnosis of Major Depressive Disorder based on DSM-5 criteria.

### 2.5. Data Analyses

To evaluate homogeneity, the under-reactive and over-reactive groups were compared using independent social and anagraphic variables and with the dependent variable (GCI score) at baseline, using χ^2^ for categorical variables and an analysis of variance for continuous variables. To test the hypotheses, we applied a repeated measures ANOVA to a group (under-reactive vs. over-reactive) and time (beforehand, 4 measurements during treatment, and after the end of the treatment) as independent variables and the CGI scores as dependent variables. Moreover, to evaluate the differences in outcomes between the two groups, χ^2^ analyses were performed on the outcome classifications (recovered, partially recovered, and/still depressed). Finally, in order to evaluate the relationships between the presence of parental psychopathology and the severity of children’s disorders at the follow-up measurement, separately in both under- and over-reactive groups, Kruskal-Wallis tests were performed with subgroups as an independent variable (children without parents with a psychiatric disorder, children with one parent with a psychiatric disorder, and children with both parents with a psychiatric disorder) and CGI score at the end-point as a dependent variable. Subsequently, the Mann-Whitney tests were performed as post hoc tests to determine the specific differences between subgroups.

## 3. Results

**Figure 1:**
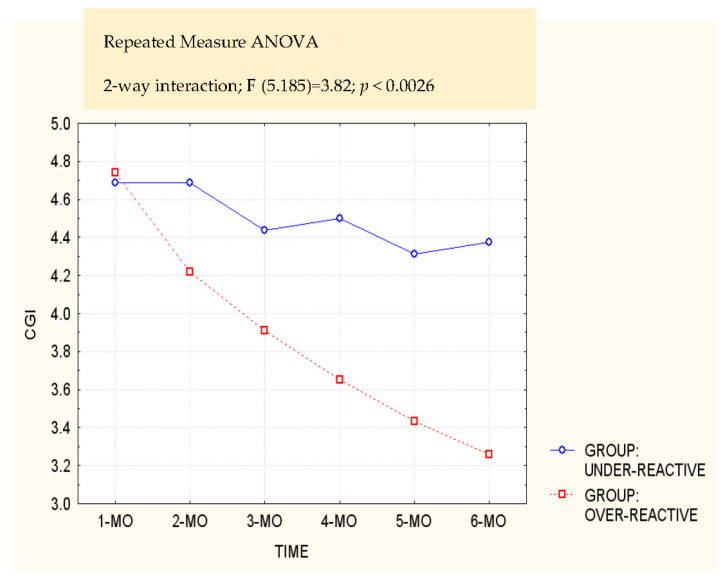
CGI mean score in the 4 measurements during treatment.

A total of 32 children and their parents were selected. Out of a total of 32 children, only 3 children (9.37% of the sample) had no parents with a psychiatric disorder, 20 children (62.50%) had at least one parent with psychopathology, and 9 (28.13%) had both parents with a psychiatric disorder. Specifically, 29 mothers (90.62% of the sample) presented a psychiatric disorder (comprising 20 subjects with a depressive disorder, 7 with an anxiety disorder, and 2 with an alcohol/substance dependence), and 9 fathers (28.13% of the sample) presented a psychiatric disorder (comprising 4 subjects with a depressive disorder, 4 with an alcohol/substance dependence, and 1 with a bipolar disorder). All the subjects of the clinical sample fell within a middle- to upper-middle-class socio-economic level, with a mean score of 71.01 (SD = 15.15) on the Hollingshead scale (1957). The under-reactive and over-reactive groups did not differ significantly in any independent variable or in a dependent variable at baseline (Table 1). Regarding the repeated measures ANOVA, group (under/over-reactive) × time (before treatment/4 measurements during treatment/after the end of the treatment) interaction was significant for the CGI variable (F = 4.11; *p* = *0*.001), and differences were all in the expected directions (Figure 1).

Specifically, post hoc tests showed a significant difference from before the treatment to after the treatment only in the over-reactive group (*p* = 0.00). Moreover, differences between the two groups were always significant in the 4 measurements during the treatment period (at 1 month: *p* = 0.05; at 2 months: *p* = 0.00; at 3 months: *p* = 0.00; at 4 months: *p* = 0.00) and in the measurement after the treatment (*p* = 0.00) (Figure 1). Regarding the outcomes, χ^2^ analyses showed a significant difference between the two groups (χ^2^ = 8.59; *p* = 0.01): in the under-reactive group, no children showed complete remission of symptoms, 5 (31.25%) were partially recovered, and 11 (68.75%) were still depressed at the follow up; in the over-reactive group 9 (39.13%) children showed complete remission of symptoms, 6 (26.09%) children were partially recovered, and 8 (34.78%) were still diagnosed as depressed at the follow-up (Table 2).

Regarding parental psychiatric disorders, in the over-reactive group, only 3 children had no parents with psychopathology, while 13 children had at least one parent with a psychiatric disorder, and 7 children had both parents with a psychiatric disorder. Kruskal-Wallis analysis showed significant differences among the 3 subgroups on children’s CGI scores at the follow-up (χ^2^ = 9.01; *p* = 0.007). Specifically, post hoc analysis using Mann-Whitney tests showed higher scores in the group with both parents with psychopathology than the groups with no parents (U = 0.00; *p* = 0.01) or one parent with psychopathology (U = 17.50; *p* = 0.02). In the under-reactive group, no child lacked a parent with psychopathology, while 7 children had at least one parent with a psychiatric disorder and 9 children had both parents with a psychiatric disorder. The Mann-Whitney analysis was thus performed comparing 2 subgroups: children with one parent with psychopathology and children with both parents with psychopathology. Analysis showed no significant difference between the subgroups on the children’s CGI scores at the follow-up (U = 17.00; *p* = 0.06). Table 3 shows the mean CGI scores and SD among the 3 subgroups, separately for the over and under-reactive groups. Table 4 shows the prevalence of maternal and paternal psychiatric disorders in the subgroups.

## 4. Discussion

Our results partly confirm data from the literature but also suggest new considerations. The over-reactive group showed a better prognosis than the under-reactive group. In fact, a significant improvement after the treatment was evident only in the over-reactive group (9 children showed complete remission). In the under-reactive group, however, no children showed complete remission of symptoms. Hence, these data confirmed that the internalizing subgroup showed a more relevant impairment in all domains compared to the over-reactive group. Moreover, the internalizing subgroup was a particularly high-risk group because of the under-identification of the need for clinical services. Hence, in our clinical sample, an over-reactive pattern confirms the mitigation of impairments in specific areas of functioning. The clinical implications related to the characteristics of different subgroups suggest the importance of direct observation of the child’s behavior in free play or during a dyadic interaction with the primary caregiver as an important part of the clinical assessment [31]. Regarding parental psychiatric disorders, in the over-reactive group, only 3 children had no parents with psychopathology, while 13 children had at least one parent with a psychiatric disorder, and 7 children had both parents with a psychiatric disorder. In the under-reactive group, all children had at least one parent with psychopathology, and 9 children had both parents with a psychiatric disorder. These data underline the strong association between parental psychiatric disorders and preschool depression [13,15]. A dysfunctional parent-child interaction is an important risk factor for the early onset of depressive symptoms. The relevant literature emphasized the importance of a facilitating environment and of a tuned and responsive mother who can facilitate the development of potential and hereditary tendencies of the infant and prevent emotive-behavioral disorders [32]. Several studies have suggested that treating the depressed mother results in the improvement of depression in the child [33]. Other investigations fail to find immediate gains after maternal treatment but detect improvements in the child’s symptoms at 6 and 12 months after remission [34]. Paternal depression should be considered. Several studies report that paternal depression 3 years after childbirth predicted later depressive symptoms, but the reverse was not true. These findings suggest that paternal depressive symptoms confer a risk for preschool depression [35]. In our clinical sample, we have considered parental psychiatric disorders, and we have found that 29 mothers (90.62% of the sample) showed a psychiatric disorder, and 9 fathers (28.13% of the sample) presented a psychiatric disorder. Considering the two subtypes, under-reactive and over-reactive, there is a more relevant correlation between maternal psychiatric pathology and the child’s internalizing psychopathological disorder. Paternal psychopathology, on the other hand, is equally distributed in the two groups (Table 4). Hence, maternal psychopathology seems to be linked to a more severe presentation of preschool depression (under-reactive group). However, it is important to underline that our results showed higher CGI scores in the group with both parents with psychopathology than the groups with no parents or one parent with a psychiatric disorder. Thus, these data highlight the importance of studies that involve fathers in clinical trials. Our data demonstrate a more relevant remission of depressive symptoms in the over-reactive group after parental support treatment. The literature suggests the importance of parent training to improve child depression. Importantly, the main objective of parent training was to teach the caregiver to act as the child’s emotion coach and external emotion regulator, implementing a theoretical model according to which early childhood depression is a disorder of emotional development. This treatment provides parents with new techniques facilitating their ability to validate rather than minimize the child’s emotional expression. The evidence suggested that parent training plays a significant role in reducing parental depression and parenting stress and encouraging children’s emotion regulation and use of reparative prosocial skills [36]. The focus of the support treatment of our clinical sample was on parental strategies to help the child manage any highly emotional experience. Hence, it seems a valid support, especially for the over-reactive group, which needs an external regulation of emotional states. The improvement of children’s depressive symptoms thus seems to be strongly related to parenting style improvement [37,38,39].

## 5. Conclusions

Hence, the observed greatest improvement in the over-reactive group could be related to the fact that this subgroup is characterized by a pattern of child emotional dysregulation, which seems to be mitigated by a more functional parenting style. Moreover, a network of environmental support involved during parental treatment may have had an important role. These considerations suggest the importance of early recognition of preschool depression in order to plan an appropriate intervention immediately and to prevent psychopathological consequences in childhood and adolescence.

## Figures and Tables

**Table 1:** Characteristics of the samples (over-reactive vs. under-reactive groups).

Variables	Over-Reactive Group N = 23	Under-Reactive Group N = 16	F/χ^2^	P
Age (in months): mean; SD	58.52; 7.65	58.31; 4.07	0.01	n.s.
Gender: boys	N = 14 (60.87%)	N = 8 (50%)	0.45	n.s.
SES: mean; SD	78.82; 11.17	75.86; 12.00	0.00	n.s.
CGI score at baseline: mean; SD	4.75; 0.45	4.70; 0.47	0.13	n.s.

**Table 2:** Outcome (over-reactive vs. under-reactive groups).

	Total Remission	Partial Remission	Absence of Remission
Over-reactive group	9 (39.13%)	6 (26.09%)	8 (34.78%)
Under-reactive group	0	5 (31.25%)	11 (68.75%)

**Table 3:** Mean and SD for the CGI score at follow-up for the subgroups with and without parental psychiatric disorder, separately for the over and under-reactive groups.

	CGI Mean; SD
*Over-reactive group.*	
Children **without** parents with a psychiatric disorder	1.67; 0.58
Children with **one** parent with a psychiatric disorder	2.85; 1.41
Children with **both** parents with a psychiatric disorder	4.43; 0.98
*Under-reactive group.*	
Children **without** parents with a psychiatric disorder	/
Children with **one** parent with a psychiatric disorder	3.86; 1.07
Children with **both** parents with a psychiatric disorder	4.78; 0.67

**Table 4:** Prevalence of parental psychiatric disorders (over-reactive vs. under-reactive group).

	Mothers with Psychiatric Disorders	Fathers with Psychiatric Disorders
Under-reactive group	81%	33%
Over-reactive group	62%	29%

## Data Availability

Data is contained within the article.
